# Utility of pT3 substaging in lymph node-negative urothelial carcinoma of the bladder: do pathologic parameters add to prognostic sub-stratification?

**DOI:** 10.1007/s00345-021-03697-3

**Published:** 2021-04-21

**Authors:** Moritz Maas, Johannes Mischinger, Eva Compérat, Marcus Scharpf, Falko Fend, Tilman Todenhöfer, Arnulf Stenzl, Georgios Gakis, Steffen Rausch

**Affiliations:** 1 Department of Urology, University Hospital, Tübingen, Germany; 2 Department of Urology, University Hospital, Graz, Austria; 3 Department of Pathology, University Hospital, Wien, Austria; 4 Department of Pathology, University Hospital, Tübingen, Germany; 5 Clinical Trials Unit, Studienpraxis Urologie, Nürtingen, Germany; 6 Department of Urology, University Hospital Würzburg, Oberdürrbacher Straße 6, 97080 Würzburg, Germany

**Keywords:** Muscle-invasive bladder cancer, Pathological staging, Patient outcome, Perivesical extension, Tumor invasion front

## Abstract

**Purpose:**

The value of bladder cancer (BC) substaging into macroscopic (pT3b) and microscopic (pT3a) perivesical fat extension in lymph node (Ln)-negative patients is controversially discussed and limited evidence for prognostic relevance of additional histopathological factors in pT3 BC exists. We evaluated the prognostic value of pT3 substaging and established pathological and clinical parameters with focus on tumor invasive front (TIF) and tumor size.

**Methods:**

Specimens of 52 patients treated with radical cystectomy (RC) for pT3 a/b muscle-invasive BC were reviewed and re-evaluated by a pathologist specialized in uropathology. Clinical variables and standard histopathologic characteristics were assessed including TIF and tumor size. Their value as prognosticators for overall survival (OS) and recurrence-free survival (RFS) was evaluated.

**Results:**

Mean age of patients was 67.55 years. Tumors were staged pT3a in 28 patients (53.8%) and pT3b in 24 (46.8%). Median OS was 34.51 months. Median tumor size was 3.2 cm, median TIF was 11.0 mm. Differences in OS between pT3a and pT3b were not significant (*p* = 0.45). Carcinoma in situ (CIS) and lymphovascular invasion (LVI) were significantly associated with pT3b tumors. Univariate analysis could not identify pathological prognosticators like TIF or tumor size for OS and RFS (*p* for all > 0.05).

**Conclusion:**

No significant differences in OS or RFS were observed comparing Ln-negative pT3 BC following radical cystectomy. Additional pathologic variables like TIF could not be identified as prognosticator. Relevance of pT3 BC substaging needs reevaluation in larger prospective cohorts.

## Introduction

Bladder cancer (BC) is one of the ten most common malignancies worldwide and the second most common uro-oncological entity [1]. Urothelial carcinoma represents the largest proportion; about 30% of the diseases are muscle-invasive at the time of their diagnosis or will become muscle-invasive in the course of their progress [2].

In patients with muscle-invasive urothelial bladder cancer (MIBC), radical cystectomy with bilateral lymphadenectomy is the treatment of choice for patients with non-metastatic disease [2]. The removed surgical specimen is pathologically examined and classified according to the American Joint Committee on Caner (AJCC) TNM staging system. This classification helps the treating physician to evaluate the disease, decide on necessary treatment options (e.g. need for adjuvant therapies) and to estimate the prognosis.

One important aspect of this classification is the pathologic tumor stage (pT). According to the currently applied classification, tumors extending the bladder without invading surrounding organs are separated into pT3a and pT3b. In this context, pT3a tumors show a microscopic and pT3b a macroscopic invasion of the perivesical fat. The prognostic significance of this distinction remains subject of controversial debates: In comparison to pT2 tumors, pT3 disease including both, pT3a and pT3b is associated with impaired prognosis [3]. However, this prognostic relevance has not consistently been reported for the comparison of pT3a and pT3b tumors, which led to discussion about the clinical importance of the pT3 sub-classification [4].

Among other clinicopathological variables with putative impact on the prognostic relevance are tumor size or the histopathological tumor invasion front (TIF) which was earlier discussed as a discriminative variable in pT3 bladder cancer [5].

Therefore, the aim of our study was to evaluate the prognostic value of pT3 substaging in dependence of additional histopathological variables including TIF and tumor size in patients with Ln-negative MICB.

## Patients and methods

From our institutional bladder cancer database, medical records of patients with diagnosis of pT3a/b MIBC that underwent radical cystectomy from 2004 to 2013 were identified. Patients with other than urothelial cancer, lymph-node metastases, or unknown nodal status were excluded from the analysis.

Files and original pathology reports were reviewed for clinical and pathologic standard variables tumor category (pT) and grade (G), lymphovascular invasion (LVI), concomitant carcinoma in situ (CIS), microvascular invasion (V1), surgical margin status (R0/1). Staging and grading was performed according to the AJCC TNM system (2015 version), and the World Health Organization staging system, respectively. All pathology specimens were re-evaluated by one uropathology specialist (E.C.) and additional pathologic parameters tumor size and tumor invasion front, defined as the maximum width of the tumor invasion front in the perivesical fat, were evaluated for the final analysis. Follow-up of patients after surgery was performed in an outpatient setting according to the respective contemporary guideline (European Association of Urology Guidelines; S3-Guideline of the German Society of Urology) recommendations. All included patients could be followed up for tumor recurrence and death.

Chi-square tests were performed to assess the association of individual clincopathological risk-factors. For categorical analyses, tumor size and TIF were grouped according to their median values. Kaplan–Meier analyses were performed to evaluate overall survival (OS) and recurrence-specific survival (RFS). Differences between subgroups were evaluated using Log-rank test. Statistical significance was regarded as *p* < 0.05. For statistic analysis, commercial software (MedCalc^®^; Version 12.7.3.0) was used. Written informed consent was obtained by all participants. The study was approved by the Ethics Committee of the University of Tübingen (Approval Number: 417/2010 A).

## Results

We identified 52 node-negative patients with pT3a/b urothelial cancer that underwent radical cystectomy and lymphadenectomy. Of the patients, 21.2% were female and 78.8% were male. One (1.9%) of the patients received neo-adjuvant chemotherapy prior to radical cystectomy, two patients (3.8%) received adjuvant platinum-based chemotherapy. The median age at surgery was 67.55 years (range 46.43–88.99). Median follow-up was 33.74 months (mean: 51.8; range 1–184.5). Of all individuals with stage pT3, 53.8% were staged pT3a and 46.2% pT3b. Median tumor size was 3.2 cm (95% CI 2.6–3.5% cm) and median TIF was 11.0 mm (95% CI 7.0–15.53 mm). Detailed patient and tumor characteristics are summarized in Table 1.

**Table 1:** Patient characteristics (*n* = 52)

Variable	*N*	%
Gender		
Male	41	78.8
Female	11	21.2
Age (years) at surgery (Radical Cystectomy)		
Median (range)	67.55 (46.43–88.99)	
Pathological stage		
pT3a	28	53.8
pT3b	24	46.8
Pathological grade		
G2	15	29.4
> G2	36	70.6
N/A	1	–
Carcinoma in situ (CIS)		
No	35	67.3
Yes	17	32.7
Lymphovascular invasion (LVI)		
No	32	61.5
Yes	20	38.5
Necrosis		
No	44	84.6
Yes	8	15.4
Vascular invasion (V1)		
V0	45	88.2
V1	6	11.8
N/A	1	–
Surgical margin status		
R0	50	96.2
R1	2	3.8
R2	0	–
Tumor size (cm)		
Median	3.2 cm (95% CI 2.6–3.5)	
Arithmetic mean	3.35 (95% CI 2.8–3.85)	
Tumor invasion front (TIF) in mm		
Median	11 (95% CI 7–15)	
Arithmetic mean	12.7 (95% CI 10–15.5)	

Overall, 21 patients (40.4%) had cancer recurrence, 33 (63.5%) patients died during follow-up. Table 2 provides survival data for the analyzed patient cohort.

**Table 2:** Tabular summary of survival data for the analyzed cohort

Patients with recurrence	*n* = *21*
Location of recurrence	
Osseous	*n* = 4
Pulmonary	*n* = 2
Local recurrence	*n* = 5
Hepatic	*n* = 2
Lymphonodal recurrence	*n* = 4
Peritoneal carcinomatosis	*n* = 2
Renal recurrence	*n* = 1
Soft tissue	*n* = 1

Death	*n* = 33
Cause of death	
Death after recurrence	*n* = 17
postoperative complications	*n* = 2
non-cancer related deaths	*n* = 14

Median overall survival (OS) was 34.51 months (24.762–89.458) for the entire cohort and 60.07 months (30.443–134.483) for pT3a patients, while the detected OS for pT3b patients accounted for 33.62 (17.800–89.458) months. The observed differences in OS were however not statistically significant. In addition no correlation between pT3 substage and RFS was identified (data not shown). Association of pT3a/b substage to the individual pathologic factors revealed CIS and LVI to be significantly associated with pT3b (Table 3).

**Table 3:** Association of pathological variables with pT3 substaging

Variable	*P*
Grade > 2	0.7508
LVI	**0.0329**
Necrosis	0.1634
CIS	**0.0472**
Positive surgical margin	0.5405
TIF > 11 mm	0.2912
Tumor size > 3.2 cm	0.1624
V1	0.7782

*LVI* lymphovascular invasion, *CIS* carcinoma in situ, *TIF* tumor invasion front, V1 vascular invasion

*p*-values in bold indicate statistical significance

**Fig. 1:**
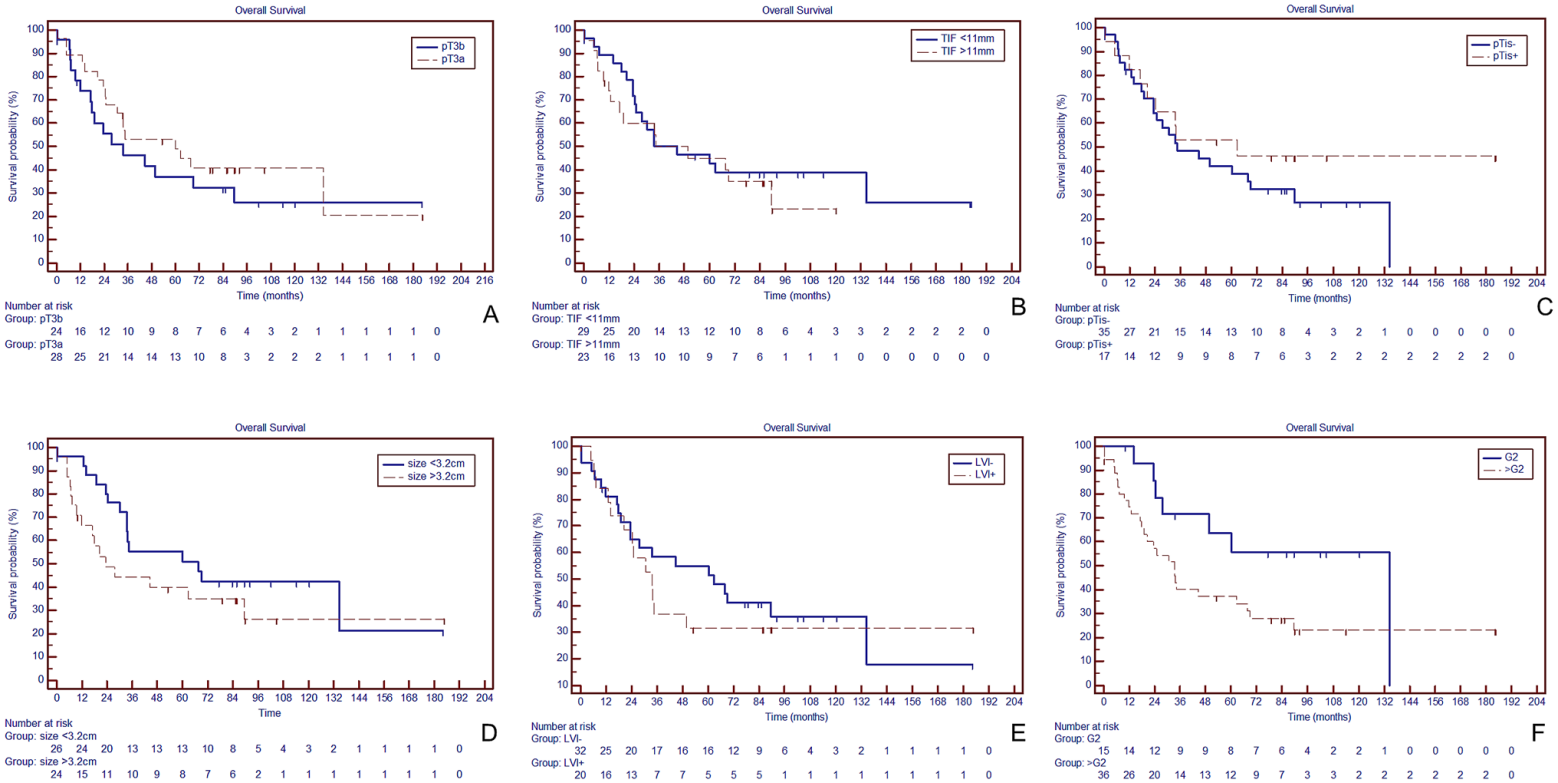
Overall survival in dependence of pT3a/b substaging and pathological variables. (*TIF* tumor invasion front, *LVI* lymphovascular invasion, *G* grade); all *p*  >  0.05

Overall, univariate analysis could not identify additional prognosticators of OS in the pT3a/b subgroup as shown in Fig. 1.

## Discussion

The current AJCC TNM classification distinguishes bladder tumors into pT3a (microscopic perivesical tissue invasion) and pT3b (macroscopic perivesical tissue invasion) sub-stages. Taking anatomical and biological aspects into account, this subdivision is evident and one would expect a worse prognosis for tumors with macroscopic invasion of the surrounding perivesical tissue: The lymphatic vessel topography shows larger lymphatic vessels more distant from the bladder’s serosa, resulting in a higher probability of lymphonodal spread in pT3b tumors [6, 7]. Corresponding observations were made by Neuzillet et al. and Tilki et al. showing more frequent LN invasion in pT3b tumors than in pT3a tumors [8, 9]. Furthermore, a macroscopic invasion is usually associated with a higher tumor burden, which leads to an increased probability of phenotypic mutations for metastatic spread and a higher probability of circulating tumor cells, capable of forming metastases [9, 10].

Despite these theoretical considerations, the expected prognostic difference between pT3a and pT3b tumors has not been consistently observed in the literature and thus leads to a discrepancy between the existing staging classification and its prognostic implication. Particularly in patients with pT3 pN0 disease no clear conclusion regarding the prognostic significance of the sub-classification is evident:

A large multicenter study by Tilki et al. shows significant differences in cancer-specific survival (CSS) and recurrence-free survival (RFS) for 456 patients with pT3, pN0 bladder cancer [8]. These observations are supported by Sonpavde et al.: Their multicentric analysis of 578 patients shows the prognostic potential of the pT3a/b sub-classification for RFS and postulates a prognostic risk model for patients with pT3 pN0 based on T3 substage at radical cystectomy, lymphovascular invasion and margin status [11]. Important limitations of the aforementioned studies are caused by their multicenter nature. Here, the pathology was not centrally reviewed and they cover long periods of time, possibly including different surgical and pathological approaches.

Conversely, a number of smaller, mainly monocentric studies in the mentioned subgroup pT3 pN0 revealed no prognostic impact of the pT3 sub-classification: Boudreaux et al. could not find any significant difference concerning OS, CSS and RFS between lymph node-negative pT3a and pT3b tumors (*p* = 0.79; *p* = 0.21 and *p* = 0.53, respectively) [12]. Similar observations were made by Dincel et al., Kim et al. and Quek et al. [13–15]. Scosyrev et al. retrospectively evaluated the SEER database (Surveillance, Epidemiology, and End results) and found impaired survival rates of pT3a BC as compared to pT2b disease with the same nodal status. Furthermore, they revealed an increased risk of nodal metastases with increased substaging and a decreased survival in pT3b patients compared to pT3a patients for node-positive tumors. However, in the node-negative subgroup, pT3a/b substage did not significantly impact OS [3]. Table 4 summarizes the available evidence on the prognostic impact of substaging in lymph node-negative pT3 patients.

**Table 4:** Summary of published data on the prognostic relevance of pT3a/b bladder cancer substaging

Study	Patients			Design	Centers	Difference OS	Difference CSS	Difference RFS	Median/mean FU in months	Remarks	Central pathological review	References
	Total	pT3a	pT3b									
Dincel et al. 2013	74	35	39	Retrospective	Monocentric	*p* = 0.938	*p* = 0.539	Not available	20.3 ± 16.9 (mean)		Yes	[13]
Kim et al. 2015	101	43	58	Retrospective	Monocentric	*p* = 0.658	*p* = 0.840	Not evaluated	32.5 (median)		Yes	[14]
Boudreaux et al 2008	75	46	29	Retrospective	Monocentric	*p* = 0.79	*p* = 0.21	*p* = 0.53	25.3 (median)		Yes	[12]
Quek et al 2004	236	35	95	Retrospective	Monocentric	*p* = 0.65	Not evaluated	*p* = 0.19	106.8 (median)	Entire study: pN1 patients included	Yes	[15]
Neuzillet et al. 2012	327	88	68	Retrospective	Monocentric	***p = 0.008***	***p = 0.01***	***p = 0.04***	23.0 (median)	Entire study: pN1 patients and pT2b patients included	Yes	[9]
Tilki et al. 2010	808	199	257	Retrospective	Multicentric	Not evaluated	***p = 0.048***	***p = 0.020***	45.0 (median)	Entire study: pN1 patients included	No	[8]
Scosyrev et al. 2010	2342	655	234	Retrospective	Multicentric	*p* = 0.78	Not evaluated	Not evaluated	Not available	Entire study: pN1 patients and pT2b patients included	No	[3]
Sonpavde et al 2011	578	356	222	Retrospective	Multicentric	Not evaluated	Not evaluated	***p < 0.0001***	39.4 (median)	Study focus on predictive model for recurrence	No	[11]

The results of our study are consistent with the reported studies above: We could not prove any negative prognostic influence of pT3b substaging compared to the pT3a patients. Moreover, given the assumption that pT3 disease may be pathologically classified and sub-stratified by alternative pathological variables, we sought to evaluate tumor size and TIF. As for the other standard pathological factors, these parameters were not shown to be prognosticators in the present analysis.

In a comparative approach, Zarei et al. evaluated the prognostic value of the depth of tumor invasion into perivesical fat in pT3 tumors and demonstrated a significant improved CSS in patients with less than 4.5 mm invasion (*p* = 0.02) [5]. A combination of both parameters, width and depth of tumor invasion, could provide new insights in this context.

Our study has several limitations which have to be addressed: First, the inherent limitation of any retrospective study should be mentioned. Additionally, it has to be noted that we reviewed a relatively small cohort of patients, which is may lead to underestimation of statistical effects. A particular strength of our evaluation is the central review of the pathological specimen by an expert in uropathology, reducing a potential inter-observer bias in the pathological assessment. Moreover, the evaluated collective is a highly selected group of advanced tumors without the prognostically significant factor of pN + status. This limits the comparability to other advanced tumor collectives and impairs the correlation between RFS and OS, as there is a comparatively large number of patients with long follow-up without recurrence. It results in an increased probability of non-cancer related death.

Since the estimation of the patient prognosis is a major goal of the AJCC staging system and the previous retrospectively collected data are contradictory in their statement, a prospective randomized trial with central pathological evaluation would be desirable to clarify the necessity of a sub-classification into pT3a and b.

## Conclusion

In our study we observed no significant difference in overall survival or recurrence-free survival in the comparison of pT3a to pT3b tumors without lymph node invasion. We further could not demonstrate a prognostic value of the length of the tumor invasion front. Further prospective randomized studies are needed to assess the requirement for pathological substaging into pT3a and pT3b.

## Data Availability

Data and material available.
